# Catabolic efficiency of aerobic glycolysis: The Warburg effect revisited

**DOI:** 10.1186/1752-0509-4-58

**Published:** 2010-05-06

**Authors:** Alexei Vazquez, Jiangxia Liu, Yi Zhou, Zoltán N Oltvai

**Affiliations:** 1 Department of Radiation Oncology, The Cancer Institute of New Jersey and UMDNJ-Robert Wood Johnson Medical School, New Brunswick, NJ, 08963, USA; 2 Department of Pathology, University of Pittsburgh, Pittsburgh, PA, 15261, USA

## Abstract

**Background:**

Cancer cells simultaneously exhibit glycolysis with lactate secretion and mitochondrial respiration even in the presence of oxygen, a phenomenon known as the Warburg effect. The maintenance of this mixed metabolic phenotype is seemingly counterintuitive given that aerobic glycolysis is far less efficient in terms of ATP yield per moles of glucose than mitochondrial respiration.

**Results:**

Here, we resolve this apparent contradiction by expanding the notion of metabolic efficiency. We study a reduced flux balance model of ATP production that is constrained by the glucose uptake capacity and by the solvent capacity of the cell's cytoplasm, the latter quantifying the maximum amount of macromolecules that can occupy the intracellular space. At low glucose uptake rates we find that mitochondrial respiration is indeed the most efficient pathway for ATP generation. Above a threshold glucose uptake rate, however, a gradual activation of aerobic glycolysis and slight decrease of mitochondrial respiration results in the highest rate of ATP production.

**Conclusions:**

Our analyses indicate that the Warburg effect is a favorable catabolic state for all rapidly proliferating mammalian cells with high glucose uptake capacity. It arises because while aerobic glycolysis is less efficient than mitochondrial respiration in terms of ATP yield per glucose uptake, it is more efficient in terms of the required solvent capacity. These results may have direct relevance to chemotherapeutic strategies attempting to target cancer metabolism.

## Background

**Figure 1:**
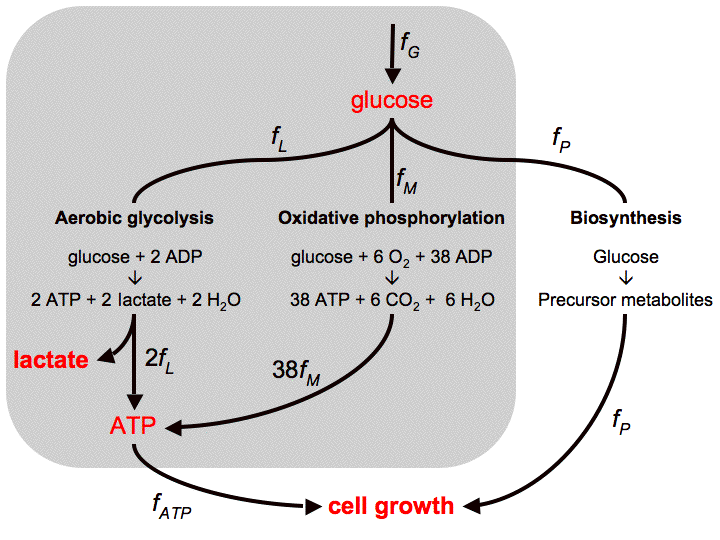
**Reduced model of cell metabolism**. Schematic representation of ATP generation pathways via aerobic glycolysis (glycolysis + pyruvate reduction to lactate) and oxidative phosphorylation (glycolysis + mitochondrial respiration). The variable *f*_*G*_, denotes the glucose uptake rate, *f*_*L *_and *f*_*M*_, the components of the glucose uptake routed towards lactate excretion and respiration, respectively, and *f*_*ATP *_the ATP production rate. Intermediate substrates of glucose catabolism are also used in a third process accounting for the production of precursor metabolites needed in anabolic processes (*f*_*P*_). The pathways considered in our model are shown in the gray-shaded area, while *f*_*P *_is treated as constant.

Since its original discovery by Warburg [1] it has been well established that most, if not all cancer cells are more dependent on aerobic glycolysis for ATP production than normal cells. The near uniform presence of this metabolic phenotype in tumor cells is counterintuitive, as glycolysis produces only 2 moles of ATP per mole of glucose, far less than the 36 generated by mitochondrial respiration (Figure 1). Several hypotheses have been proposed for the maintenance of this seemingly wasteful catabolic state. At the cell population level, mitochondrial respiration malfunction and enhancement of glycolysis are thought to be a metabolic advantage under the intermittent hypoxia conditions experienced by pre-malignant and malignant tumor cells [2,3]. However, aerobic glycolysis is not found exclusively in cancer cells, but is also observed in rapidly dividing normal cells even under conditions of normoxia [4]. It is also widely believed that the glycolysis rate increases in order to match the increased anabolic needs of the rapidly proliferating cells for precursor metabolites [5]. Yet, a focus on the cell's anabolic needs alone neglects two important facts; First, in addition to precursor metabolites, growing cells need ATP to meet the energy demands of biosynthetic pathways. Second, in its original definition the Warburg effect refers to the increase in the glycolysis rate ending in the excretion of lactate, which does not contribute to the production of precursor metabolites.

The actual molecular mechanisms that lead to the enhanced aerobic glycolysis are increasingly well understood. Under physiological condition, for example, PI3K/Akt signaling pathways play a critical role in promoting aerobic glycolysis in activated T cells in response to growth factors or cytokines stimulation [6]. In the pathophysiological condition of tumorigenesis, malfunction of mitochondrial respiration due to mitochondrial DNA mutations/deletions is an important contributing factor [7-10]. Also, p53, one of the most frequently mutated genes in cancers, is both a positive regulator of mitochondrial respiration [11] and a negative regulator of glycolysis [12]. These, together with the activation of hypoxia-inducible factor (HIF), a transcription factor that is activated by hypoxic stress but also by oncogenic, metabolic, and oxidative stress, often lead to the overexpression of the glucose transporters and various glycolysis pathway enzymes or isozyme subtypes explaining the increased glucose uptake and altered utilization [13,14].

The evidence accumulated so far thus indicates that the presence of aerobic glycolysis is a common characteristics of rapidly proliferating cells, and that it may offer a growth advantage to rapidly proliferating normal cells, e.g., during development and tissue regeneration, and to cancer cells in tumor formation. Yet, a system-level interpretation of the origin of this growth advantage has not yet been formally provided. To start addressing this issue, here we introduce a reduced flux balance model of ATP generation that incorporates a glucose uptake capacity constraint and the limited solvent capacity of the cell cytoplasm. The model allows us to uncover the existence of two different energetically favorable metabolic regimes within proliferating cells, a finding that is congruent with experimental results.

## Results

### Reduced flux balance model of cell metabolism

Figure 1 depicts a schematic model of glycolysis and mitochondrial respiration, the main pathways for ATP generation in cells. The glucose uptake flux (*f*_*G*_) is partitioned into the flux of aerobic glycolysis (*f*_*L*_), and to oxidative phosphorylation (*f*_*M*_). Aerobic glycolysis represents glycolysis, converting glucose into pyruvate, and then pyruvate reduction by lactate dehydrogenase (LDH) in the cytosol, resulting in the end product lactate that is then excreted to the extracellular millieau. ATP generation through oxidative phosphorylation is decomposed into the generation of pyruvate through glycolysis, followed by the oxidation of pyruvate in the TCA cycle and the respiratory chain, the latter two processes taking place in the cell's mitochondria. There is also a third component for glucose utilization accounting for the production of precursor metabolites needed in anabolic processes (*f*_*P*_) (e.g., intermediate metabolites of the pentose phosphate pathway and the TCA cycle [5]), which have to have an overall proportionality with the available energy currencies (phosphate donors, mainly ATP) to enable cell growth. Thus, in the absence of a full scale kinetic model, in our modeling we assume that the ATP production rate (*f*_*ATP*_) is proportional with the rate of anabolic processes (*f*_*P*_), and use *f*_*ATP *_as a surrogate for the overall cell metabolic rate (Figure 1).

Aerobic glycolysis includes the glycolysis pathway producing pyruvate and the pyruvate reduction producing lactate. The overall reaction for aerobic glycolysis is(1)

Aerobic glycolysis has a yield of 2 moles of ATP per mole of glucose, resulting from the conversion of glucose into lactate. On the other hand, oxidative phosphorylation includes the glycolysis pathway producing pyruvate, the oxidation of pyruvate in the TCA cycle, and the respiratory chain. The overall reaction of for oxidative phosphorylation is(2)

Oxidative phosphorylation yields 38 moles of ATP per mole of glucose, two of which are produced in glycolysis and the other 36 during the oxidation of pyruvate in the TCA cycle coupled to respiratory chain activity. Summing up these differential contributions we obtain the rate of ATP production(3)

where the second equality was obtained using *f*_*G *_= *f*_*L *_+ *f*_*M*_.

Our aim is to determine the optimal flux distribution (*f*_*L*_, *f*_*M*_) that provides maximum ATP production rate given the cell's metabolic constraints. Of these, the first metabolic constraint is associated with the maximum glucose uptake rate (or glucose uptake capacity)(4)

where *F*_*G *_is the maximum glucose uptake rate. The second constraint, quantified below, reflects on the high concentration of macromolecules within the cell's cytoplasm [15,16], resulting in a limited solvent capacity for the allocation of metabolic enzymes. Enzyme molecules have a finite volume and the total sum of their volumes cannot exceed the cell volume. In our case, this constraint applies to the volume occupied by glycolytic enzymes, LDH and mitochondria. Enzymes associated with other pathways occupy a fraction of the intracellular volume as well. Nevertheless, this fraction simply restricts the amount of the cytoplasmic space available to glycolytic enzymes, LDH and mitochondria. More precisely, if *V*_*G*_, *V*_*L *_and *V*_*M *_are the cell volume occupied by glycolytic enzymes, LDH and mitochondria, respectively, then(5)

where *V*_*ATP *_is the total cell volume reserved for the allocation of components of the ATP producing pathways. The occupied volumes *V*_*G*_, *V*_*L *_and *V*_*M *_are proportional to the enzyme masses *M*_*G*_, *M*_*L *_and *M*_*M*_, with *V*_*G *_= *v*_*G*_*M*_*G*_, *V*_*L *_= *v*_*L*_*M*_*L *_and *V*_*M *_= *v*_*M*_*M*_*M*_, where *v*_*G*_, *v*_*L *_and *v*_*M *_are the specific volumes of glycolytic enzymes, LDH and mitochondria, respectively. In turn, the glycolytic rate (*f*_*G*_), the lactate excretion rate (2*f*_*L*_) and the mitochondrial ATP production rate (36*f*_*M*_) are proportional to the mass of glycolytic enzymes, lactate dehydrogenase and mitochondria respectively, with *f*_*G *_= *r*_*G*_*M*_*G*_/*V*, 2*f*_*L *_= *r*_*L*_*M*_*L*_/*V *and 36*f*_*M *_= *r*_*M*_*M*_*M*_/*V*, where *r*_*G *_is the glycolytsis rate per unit of glycolytic enzyme mass, *r*_*L *_is the rate of lactate production per mass of LDH, *r*_*M *_is the mitochondrial ATP production rate per unit of mitochondrial mass, and *V *is the cell volume. In these equations the product by the mass and the division by the cell volume takes into account that the rates *r *are commonly reported in the literature per unit of dry weight, while the pathway rates *f *are reported per unit of cell volume. Because of the interdependency of volume, mass and reaction rate, the volume constraint (5) can be translated to the metabolic constraint(6)

where *ϕ *_*ATP *_= *V*_*ATP*_/*V *is the total volume fraction of the cell cytoplasm occupied by glycolytic enzymes, LDH and mitochondria, and the *crowding coefficients a*_*G *_= *v*_*G*_/*r*_*G*_, *a*_*L *_= 2 *v*_*L*_/*r*_*L *_and *a*_*M *_= 36 *v*_*M*_/*r*_*M *_quantify the occupied volume fractions per unit of glycolytic, lactate excretion and mitochondrial respiration rate, respectively. Using empirical data reported in the literature we have estimated the crowding coefficients (see Methods). We obtain *a*_*G *≈ _0.0027 (min/mM), *a*_*L*≈ _0.00023 (min/mM) and *a*_*M *_≈ 0.10 (min/mM), indicating that the mitochondria contributes about 5 and 50 times more to molecular crowding than glycolytic enzymes and lactate dehydrogenase, respectively. The unexpected consequences derived from this fact will be uncovered below.

In our modeling we assume that *V*_*ATP*_, *r*_*G*_, *r*_*L *_and *r*_*M *_are constant parameters that can be obtained from experimental estimates. Note though, that this is an approximation, as there may be regulatory mechanisms that under certain environmental or developmental conditions are capable of altering the amount of intracellular space allocated to ATP producing pathways and the activity of glycolytic enzymes, LDH and mitochondria.

Taken together the relevant metabolic optimization problem is as follows: maximize the ATP production rate (3) under the metabolic capacity constraints (4) and (6).

### Optimal solution

In the following we discuss the model-predicted utilization of aerobic glycolysis and oxidative phosphorylation pathways in the context of the cell's glucose uptake rate. It becomes obvious that the glycolysis rate matches the maximum glucose uptake capacity (*f*_*G *_= *F*_*G*_) to maximize the ATP production rate. However, there are some differences in the flux distribution along aerobic glycolysis and oxidative phosphorylation depending on the glucose uptake rate. Specifically,(7)

for *f*_*G *_<*f*_1_, and for *f*_*G *_≥ *f*_1_(8)

where(9)(10)

**Figure 2:**
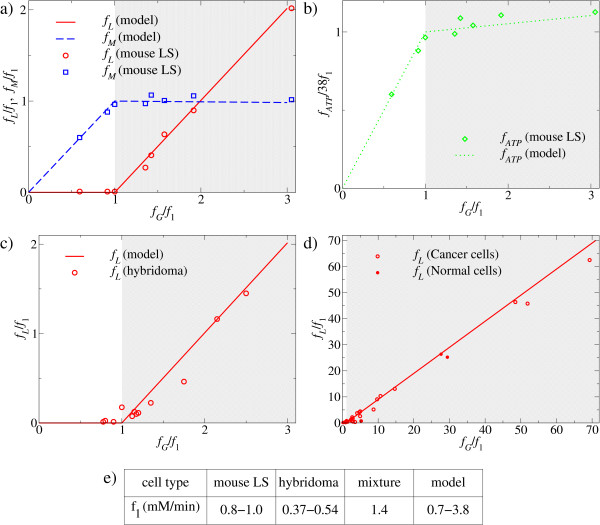
**Catabolic regimes within proliferating mammalian cells**. a, b, c, d) Model-predicted (lines) and measured (symbols) fluxes as a function of the glucose uptake rate: a) and b) LS mouse cells, c) hybridoma cells and d) mixture of cancer and normal cells. The two different metabolic regimes are indicated by the white and gray backgrounds, respectively. All fluxes are reported in units of *f*_1_, the glucose uptake rate threshold, reported in panel e).

The optimal solution is graphically illustrated in Figure 2a and 2b (lines). At low glucose uptake rates ATP is entirely produced by the oxidative phosphorylation pathway and there is no lactate production (and excretion). This trend continues up to a threshold glucose uptake rate, *f*_1 _(9), when mitochondria occupy the entire cell volume fraction (*V*_*ATP*_) available for ATP production pathways (Figure 2a, blue dashed line, white background, *glucose-limited regime*). Beyond this threshold value the concentration of mitochondria, and therefore the rate of ATP production through oxidative phosphorylation, cannot be increased further. However, additional glucose uptake may be diverted toward pathway(s) less efficient in terms of ATP yield per mole of glucose. Indeed, the optimal solution predicts a metabolic switch, in which glucose uptake rates larger than *f*_1 _leads to a linearly increasing lactate production (Figure 2a, red solid line, gray background, *space-limited regime*), and therefore a component of the ATP production is now derived from aerobic glycolysis. Furthermore, the increase in lactate production is accompanied by a slight, gradual reduction of the oxidative phosphorylation flux (Figure 2a, blue dashed line, gray background). Therefore, due to the increased aerobic glycolysis rate ATP production can still be increased beyond *f*_1 _(Figure 2b, green dotted line). However, because aerobic glycolysis has a lower yield than oxidative phosphorylation, the ATP production rate increases with a lower slope beyond *f*_1_. Finally, it is worth noting that because *a*_*M *_is 5 and 10 times higher than *a*_*G *_and *a*_*L*_, respectively, these plots are not too sensitive to the precise estimates of the crowding coefficients. Indeed, assuming that *a*_*M *_is much greater than *a*_*G *_and *a*_*L*_, we can approximate (8) by *f*_*M *≈ _*f*_1 _and *f*_*L *≈ _*f*_*G*_-*f*_1_. Therefore, we predict that the plots of *f*_*M *_and *f*_*L *_versus *f*_*G *_are rather universal, with a distinctive change of behavior at *f*_*G *_= *f*_1_.

### Experimental evidence for model validity

The model described above results in two experimentally testable predictions: the glucose uptake threshold *f*_1 _(9), where the metabolic switch takes place, and the shape of the lactate excretion, oxidative phosphorylation and ATP production plots as a function of the glucose uptake rate. To start addressing the validity of these predictions we first utilized previously reported experimental data for mouse LS cells [17] (Figure 2a, b), hybridoma cells [18] (Figure 2c), and a mixture of cancer and normal cells [4] (Figure 2d), indicating that cancer cells and normal cells follow the same law.

In the first two cases [17,18], the growth rate of the cells were manipulated by their growth in chemostat cultures at different dilution rates, a protocol that is known to substantially vary the cells' glucose uptake rate. In both cases the empirical lactate excretion plots (Figure 2a, c, red circles) follow the model predictions (Figure 2a, c, red solid lines): at low glucose uptake rates there is no significant lactate excretion, but once reaching a threshold the lactate excretion rate increases linearly with the further increase in glucose uptake rate. Moreover, the slope of this linear increase coincides with that predicted by the model. For the LS cells, we can also derive their oxidative phosphorylation (Figure 2a, blue squares) and ATP production rate (Figure 2b, diamonds) from the published experimental data [17] (see Additional file 1 Table S2 for details), and in both cases we obtain a good agreement between the experimental data (symbols) and the model predictions (lines). In the third case, the mixture of cancer and normal cells is a compilation of experimental reports using different methods and cell lines [4] (see also Additional file 1 Table S4). Here again, glucose uptake rates and lactate excretion levels show a good correlation to each other (Figure 2d).

The glucose uptake threshold, i.e., the uptake rate where the switch takes place, can be estimated from a linear fit to the lactate excretion plot in the regime where lactate is excreted (Additional file 2 Figure S1). It ranges between 0.8-1.0 mM/min for mouse LS cells, 0.37-0.54 mM/min for hybridoma cells and 1.4 mM/min for the mixture of cancer and normal cells (Figure 2e). Based on independent empirical estimates of the cell volume fraction occupied by mitochondria and the ATP production rate per unit of mitochondrial mass, our model predicts the threshold glucose uptake rate, *f*_1_, to be in the range between 0.7 and 3.8 mM/min (Methods), which overlaps with the ranges reported above for the different cell lines. This agreement is surprisingly good given that they were estimated using independent data.

To further test the model's predictive capability, we have next examined human BJ fibroblast cells serially transduced with the catalytic domain of human telomerase (hTERT), SV40 large T (LT) and small T (ST) antigens, and an oncogenic allele of H-ras (H-RasV^12^) [19]. Previous studies using these cells have shown, as we partially confirm here (Additional file 2 Figure S2), that the growth rate, soft agar colony formation, and in vivo tumorigenicity of CL1-[hTERT], CL2-[hTERT+LT], CL3-[hTERT+LT+ST], and CL4-[hTERT+LT+ST+H-Ras] cells largely correspond with their level of transduction [19], and that their glucose uptake rates progressively increase from normal toward more tumorigenic transformants (CL1 to CL4)) [20]. Previous experiments also demonstrated that normal CL1 cells display a severe reduction in their ATP content upon inhibition of mitochondrial oxidative phosphorylation, while the fully tumorigenic CL4 cells remain largely unaffected (CL2 ad CL3 cells display an intermediate reduction in their ATP levels) [20]. In contrast, compared to the other three cell types CL4 cells display higher level of lactate production and a more severe reduction in their ATP content when their LDH activity is inhibited [20].

To confirm and extend the above findings, we experimentally tested the extracellular lactate levels, mitochondrial mass and mitochondrial membrane potential (a proxy for respiratory chain activity) of CL1-4 cells at 48 h, 72 h, 96 h, and 120 h after seeding the same amount of cells, along with cell counts and total protein concentration measurements. We find that the tumorigenic CL4 cells have the highest extracellular lactate levels compared to the other cell types at all time points tested that correlates with a concomitant change in LDH enzyme activity but not protein expression level (Additional file 2 Figure S3). CL4 cells also have the lowest mitochondrial mass and membrane potential, though compared to CL1 cells their decrease is larger than predicted by our model (Additional file 2 Figure S4) (a detailed description of the results is given in the Additional file 2). Taken together, the observed alterations in lactate production and mitochondrial parameters, together with the previously observed oxidative phosphorylation and LDH activity inhibition profiles in the same cells [20], are congruent with the model predictions: an increase of lactate excretion and decline of mitochondrial respiration with an increasing replicative/tumorigenic capacity (quantified by the ATP production rate in the model).

## Discussion and conclusions

Rapidly proliferating mammalian cells, including cancer cells, almost always exhibit aerobic glycolysis even under normoxic conditions, characterized by an increased lactate excretion rate relative to the respiration rate. Since its original observation by Warburg [1], from the standpoint of metabolic efficiency this has been a surprising observation because aerobic glycolysis is far less efficient than mitochondrial respiration in terms of moles of ATP generated per mole of glucose. Implicit to this result, however, is the assumption that glucose uptake is a limiting factor of cell metabolism. Yet, the validity of this assumption on metabolic efficiency in the context of additional physicochemical constraints of the cell has not been formally addressed.

By developing a reduced flux balance model of ATP production (Figure 1) here we demonstrate the existence of two different metabolic regimes in mammalian cells, depending on whether the cell metabolic rate is limited by the glucose uptake or by the solvent capacity of the cell's cytoplasm. In conditions where glucose transport limits the cell's metabolic activity we find that the optimal solution is to produce ATP entirely via mitochondrial respiration, confirming that ATP generation yield is the appropriate measure within this regime; However, at high glucose uptake rates the optimal solution is characterized by an abrupt increase of the flux towards lactate production (and excretion) and a gradual decrease of mitochondrial respiration (Figure 2). This second regime is determined by the existence of a limited cytoplasmic solvent capacity for allocating the components of the ATP generating pathways (see equation (6)). For glucose uptake rates exceeding the threshold level, *f*_1_, the cell cannot further increase the concentration of mitochondria to increase the respiration rate in order to match the increased glucose uptake rate. Yet, the glucose uptake "surplus" may now be diverted towards a pathway that is less efficient in terms of ATP yield per mole of glucose but is characterized by a higher rate of lactate (and thus ATP) production per its own (i.e., glycolytic and LDH enzymes) mass than the rate of ATP production by respiration per mitochondrial mass.

Comparisons to experimental data reported for LS mouse cells [17], hybridoma cells [18] and a collection of normal and cancer cells [4] provide an indirect support for the validity of these predictions. The agreement between the experimentally determined fluxes and the model predictions is remarkably good (see Figure 2). In all cases the empirical plot of the lactate excretion as a function of the glucose uptake rate (symbols) falls close to that predicted by the model (lines). More importantly, the experimental values indicate a glucose uptake threshold around 1 mM/min, which falls in the range *f*_1_~0.7-3.8 mM/min predicted by our model. Also, experimental data reported here and previously [20] on lactate production, mitochondrial parameters and enzyme inhibition profiles of genetically modified human BJ fibroblast cells [19] are congruent with the model's predictions. In similar recent experiments, oncogenic Ras/E1A transformed low passage murine embryonic fibroblast (MEFs) (Ras-LP) also displayed higher mitochondrial respiration activity and lower lactate secretion compared to more rapidly dividing high passage MEFs (Ras-HP) [21], and tumor progression in patients may correlate with reduced mitochondrial density and/or function [22-24].

The model described here has its limitations and does not account for all the potential population-level advantages of the Warburg effect. First, decreased mitochondrial respiration also contributes to the mitochondrial depolarization that is frequently observed in tumor cells, and that is thought to dampen the cell's propensity for mitochondrially-induced apoptosis [25]. Secondly, the effect of the secreted lactic acid on the surrounding normal tissue may increase the tumor cells' invasive potential [3]. Thus, tumor cells may evolve to decrease mitochondrial respiration (and increase glycolysis) even further than predicted by our model. Third, increased glycolytic rates can increase the sensitivity of the pathways producing precursor metabolites to specific regulators, allowing high rates of proliferation when required [26]. Moreover, our simplified model focuses on the ATP demand and the cell volume fraction occupied by mitochondria. However, in addition to ATP rapidly proliferating cells need increased amount of precursor metabolites to fuel their biosynthetic pathways [5,27]. Thus, ultimately a cell-scale metabolic model will need to be developed to take into account all the cell's metabolic demands. The achievement of this goal is currently limited by the unavailability of enzyme activity measurements at the human metabolic network scale.

From a more general perspective, it is evident that aerobic glycolysis is not unique to cancer- and rapidly proliferating normal mammalian cells but is present in unicellular organisms, as well. For instance, under aerobic growth conditions fast growing *E. coli *and *S. cerevisiae *cells also exhibit aerobic glycolysis, resulting in the excretion of the metabolic byproducts, acetate and ethanol, respectively. More importantly, both for *E. coli *and for *S. cerevisiae *a shift toward aerobic glycolysis follows a switch from limited to rich nutrient conditions [28,29], and for *E. coli *the maximum growth rates in different carbon sources and the mode of utilization of mixed carbon sources are in agreement with those allowed by the limited solvent capacity of the cell's cytoplasm [29,30]. Taken together with our current finding for mammalian cells, these results suggest that the appearance of aerobic glycolysis provides an energetically favorable catabolic state for all rapidly proliferating cells due to the inherent physicochemical constraint of molecular crowding on cell metabolism.

These results may have relevance to cancer therapy as well. It has been previously proposed that the Warburg effect may represent a unique property of cancer cell metabolism [13]. Pharmacological reduction of lactate production and excretion may indeed reduce the invasive potential of tumor cells or makes them more susceptible to apoptosis [25]. However, the metabolic state of tumor cells itself is not a unique target for cancer chemotherapy, though individual, tumor specific isozymes may be targeted [14].

## Methods

### Parameters estimates: model

*a*_*G*_: The glycolysis rate per total mass of glycolytic enzyme is about *r*_*G *_= 0.29 mmol glucose uptake/min/g at 30°C [31], obtained as 23 μmol/min/mL lactate production rate (Table four [31]), divided by 40 mg/mL of total glycolytic protein concentration (Table two [31]), and divided by 2 to convert from lactate production to glucose uptake. The specific volume of glycolytic enzymes is estimated from the specific volume of globular proteins 0.79 mL/g [32], thus we use *v*_*G *_= 0.79 mL/g. Putting these two values together we obtain *a*_*G *_= *v*_*G*_/*r*_*G *_= 0.0027 (min/mM). *a*_*L*_: The rate of lactate production per mass of lactate dehydrogenase is about *r*_*L *_= 7 mmol lactate/min/g at 30°C [31], obtained as 23 μmol/min/mL lactate production rate (Table four [31]), divided by 3.2 mg/mL of lactate dehydrogenase concentration (Table two [31]). The specific volume of lactate dehydrogenase is estimated from the specific volume of globular proteins 0.79 mL/g [32], thus we use *v*_*L *_= 0.79 mL/g. Putting these two values together we obtain *a*_*L *_= 2*v*_*L*_/*r*_*L *_= 0.00023 (min/mM). *a*_*M*_: The mitochondrial rate of ATP generation per mitochondrial mass is in the range of 0.1-1.0 mmol ATP/min/g [33-35]. We use the maximum value *r*_*M *_= 1.0 mmol ATP/min/g. The mitochondrial specific volume has been reported to be 3.15 mL/g in mammalian liver [36] and 2.6 mL/g in muscle [37], respectively. We use the average between these two values *v*_*M *_= 2.9 mL/g. Putting these two values together we obtain *a*_*M *_= 36*v*_*M*_/*r*_*M *_= 0.10 (min/mM). *ϕ*_*ATP*_: The cell volume fraction occupied by cellular components involved in the generation of ATP was estimated from the cell volume fraction occupied by mitochondria. Literature reports for the latter range between 0.07 in some cell lines to 0.38 in muscle cells (Additional file 2 Table S1). Thus we use *ϕ*_*ATP *_= 0.07-0.38. *f*_1_: Substituting the experimental estimates *a*_*M *_= 0.17 (min/mM), *a*_*G *_= 0.0027 (min/mM) and *ϕ*_*ATP *_= 0.07-0.38 into (9) we obtain *f*_1 _= 0.7-3.8 mM/min.

### Parameter estimates: flux data

Murine LS cells: The experimental flux measurements for the mouse LS cell line were obtained from reference [17] (Additional file 1 Table S2), reporting the glucose uptake, lactate production and oxidized glucose rates, in units of *μ*mole per 10^6 ^cells per day. The experimental value of *f*_1 _was estimated from a linear fit to the data points in the region where there is a significant lactate production (Additional file 2 Figure S1a), resulting in *f*_1 _= 0.69 *μ*mole per 10^6 ^cell per day. Taking into account that LS mouse cells have a typical volume of 500-600 *μ*m^3 ^[38] we obtain *f*_1 _= 1 mM/min. Hybridoma cells: The experimental flux measurements for the hybridoma cell line were obtained from reference [18] (Additional file 1 Table S3), reporting the glucose uptake and lactate production rates, in units of mmole per 10^9 ^cells per hour. The experimental value of *f*_1 _was estimated from a linear fit to the data points in the region where there is a significant lactate production (Additional file 2 Figure S1b), resulting in *f*_1 _= 0.042 mmole per 10^6 ^cells per hour. Taking into account that hybridoma cells have a typical volume of 1300-1900 *μ*m^3 ^[39] we obtain *f*_1 _= 0.37-0.54 mM/min. Mixture of cancer and normal cells: The experimental flux measurements for the mixture of cancer and normal cells were obtained from reference [4] (Additional file 1 Table S4), reporting the glucose uptake and lactate production rates, in units of mmol/g/h. The experimental value of *f*_1 _was estimated from a linear fit to the data points in the region where there is a significant lactate production (Additional file 2 Figure S1c), resulting in *f*_1 _= 0.029 mmol/h/g. Assuming a typical cell density of 0.34 g/mL we obtain *f*_1 _= 1.4 mM/min.

### Experimental Protocols

Genetically-manipulated derivatives of human BJ fibroblasts, transfected with the catalytic subunit of human telomerase (hTERT) (CL1), hTERT+ SV40 large T (LT) (CL2), hTERT+LT+ SV40 small T (ST)(CL3), and hTERT+LT+ST+ V^12 ^mutant human Ras (Ras) (CL4) [19] were generous gifts from Dr. W. Hahn (Dana Farber Cancer Institute, Boston). The cells were maintained in DMEM supplemented with 25 mM glucose, 4 mM glutamine and 10% FBS at 37° in a CO_2 _incubator with 5% CO_2 _and 95% air. The experimental protocols for Western blots, Ras activity measurements cell growth and soft agar assays, LDH activity measurements, and for measuring mitochondrial mass and membrane potential are provided in the Additional file 2.

## Competing interests

The authors declare that they have no competing interests.

## Authors' contributions

AV and ZNO conceived the project. AV developed the metabolic model and analyzed the literature data. JL and YZ performed the reported experiments. AV and ZNO wrote the manuscript. All authors read and approved the final manuscript.

## Supplementary Material

Additional file 1 **Supplementary tables**. Supplementary Tables S2, S3 and S4 Click here for file

Additional file 2 **Supplementary information**. Experimental protocols and results (p2-4), Table S1 for parameter estimates (p6), Supplementary Figures (p6-8). Click here for file
