# Supplementary material for: Catabolic efficiency of aerobic glycolysis: The Warburg effect revisited
Source: BMC Syst Biol. 2010 May 6;4:58. doi: 10.1186/1752-0509-4-58 (PMC2880972; doi:10.1186/1752-0509-4-58)
Supplement: Additional file 2 — Supplementary information. Experimental protocols and results (p2-4), Table S1 for parameter estimates (p6), Supplementary Figures (p6-8). [file 1752-0509-4-58-S2.PDF]

Supplementary Information

Catabolic efficiency of aerobic glycolysis: The Warburg effect revisited

A. Vazquez[1], J. Liu[2], Y. Zhou[2], and Z.N. Oltvai[2]

[1]Department of Radiation Oncology, The Cancer Institute of New Jersey and UMDNJ-Robert Wood Johnson Medical School, 195 Little Albany St, New Brunswick, NJ 08963, USA

[2]Department of Pathology, University of Pittsburgh, Pittsburgh, PA, 15261, USA

Methods and Results for the following sections

Experimental protocols and results p2-4

Table S1 for parameter estimates p6

Supplementary Figures p6-8

References p9-10

Experimental Protocols and Results

Experimental protocols

Cell culture and growth rate determination

Genetically-manipulated derivatives of human BJ fibroblasts, transfected with the catalytic subunit of human telomerase (hTERT) (CL1), hTERT+ SV40 large T (LT) (CL2), hTERT+LT+ SV40 small T (ST)(CL3), and hTERT+LT+ST+ V^{12} mutant human Ras (Ras) (CL4) [1] were used. The cells were maintained in DMEM supplemented with 25mM glucose, 4mM glutamine and 10% FBS at 37° in a CO_2 incubator with 5% CO_2 and 95% air. For assessing their growth rate cells were seeded at a density of 2×10^4 cells in 12-well plates and at every 24 h a subset were collected following trypsinization (in 0.25% trypsin, 0.53 mM EDTA). Cell numbers were determined by blinded counting of cells on a standard grid-patterned hemocytometer.

Western blot

CL1-4 cells were harvested by trypsinization at 48h, 72h, 96h and 120h after seeding, and washed with PBS. The cell pellets were dissolved in 100µl RIPA lysis buffer supplemented with Halt complete protease inhibitor cocktail and phosphatase inhibitor cocktail (ThermoFisher) on ice for 1h. After centrifugation for 15min at 4°C, and the protein concentration of the supernatants were determined by Bradford assay (Bio-Rad). 50 µg of each sample was then applied to 15% Tris SDS-PAGE gel and, following electrophoresis, transferred to PVDF membranes at 6v for 16 hr at 4°C. The membranes were then immunostained using actin-specific monoclonal antibody (Sigma, 1:8000), H-Ras monoclonal antibody (Santa Cruz Biotech, 1:500), LDH monoclonal antibody (Santa Cruz Biotech, 1:4000) and VDAC/porin monoclonal antibody (Abcam, 1:5000), respectively; All experiments were performed in triplicates.

Ras activity measurements

Ras activity was performed using active Ras pull-down and detection kit (Thermo Scientific), as instructed. Briefly, 1mg fresh lysates from CL1-4 were subjected to affinity precipitation of activated Ras with the GST-fused Ras-binding domain (RBD) of Raf1 through the glutathione agarose resin. Half of the eluted samples (25µl, active Ras) from CL1-4 lysates were separated with 15% Tris SDS-PAGE gel and immunoblotted with anti-Ras Ab.

Soft agar assay

Soft agar assays were performed as described [2]. Briefly, 10,000 cells from each cell lines were resuspended in 2 ml of 0.35% top agar in complete DMEM growth medium and seeded in six-well plates previously filled with 2 ml of 0.7% basal agar in complete growth medium. The assay was performed in duplicate. After 2 weeks, 1ml 0.005% crystal violet was added to each well to stain the colonies for 2h and colonies in each well were counted.

LDH activity measurements

LDH activity was determined by the decreasing absorbance of β-NADH at 340nm as described [3,4] with slight modifications. Sodium pyruvate and β-NADH were dissolved separately in 0.2M Tris-HCl, pH 7.4. The mixture solution of 2mM sodium pyruvate and 0.2mM β-NADH was prepared fresh before being used in the LDH activity assay. A total

cell lysate sample (100μg) was pipetted into a 1.5ml disposable semi-micro cuvette (1cm light path), followed by the addition of 1ml pyruvate-NADH mixture solution. After mixing the cuvette was immediately placed in the spectrophotometer and the absorbance was recorded for 30s at 340nm. The slope of the absorbance over this time period (-ΔA/min) was proportional to the rate of NADH consumption, which represents the LDH activity.

Measuring mitochondrial content and membrane potential
Cells were trypsinized and washed with PBS in suspensions of 1 x 10^6 cells/ml at the indicated time points, followed by incubating them with 2μg/ml JC-1 or 100nM MitoTracker Green (Invitrogen) at 37 °C for 15min, respectively. After rinsing with PBS, cells were subjected to flow cytometry to read the fluorescence intensity at 590nm (red channel) and 520nm (green channel). All fluorescence intensities were normalized to that of CL1 fibroblasts at 48h.

Experimental results

Characterization of cell types

CL1-4 cells were grown in DMEM supplemented with 25mM glucose, 4mM glutamine and 10% FBS at 37° in a CO_2 incubator with 5% CO_2 and 95% air. Their Ras expression and activity, colony formation in soft agar, and growth rate were determined as described above, and the obtained results were similar to that previously described [1, 5] (Fig. S2).

Extracellular lactate levels and LDH enzyme activities

Since CL1-4 cells display a slightly different morphology (data not shown), we corrected for the absolute lactate production by measuring the total protein concentration of each cell line. It is evident that compared to the other cell types CL4 cells have a significantly higher extracellular lactate levels than CL1 and CL2 cells at each time point tested (Fig. S3a). Of note, starting from 96h CL3 cells also display significantly higher extracellular lactate levels, a finding that is likely related to their increased growth rate that is similar to that of CL4 cells (Fig. S2c), and that has been shown to be independent of H-Ras expression level or activity [6]. The LDH enzyme activities of CL3 and CL4 are significantly higher than that of CL1 and CL2 cells at most time points (Fig. S3b). However, LDH protein expression levels are not significantly different among the four cell types (Fig. S3c). These data confirm that the lactate production rate increases with the rising glucose uptake rates of these cells [5], and indicate that the increase in LDH activity is primarily achieved by posttranslational activation of the enzyme.

Mitochondrial parameters

We also compared the mitochondrial mass and membrane potential level (a proxy for mitochondrial respiratory activity) of CL1-4 cells as a function of time at 48h, 72h, 96h and 120h after seeding. The mitochondrial membrane potential of CL1-4 cells was assessed by staining with JC-1, a dye that is able to monitor mitochondrial membrane potential (largely) independent of mitochondrial mass. The mitochondrial content of the cell was assessed using MitoTracker Green (MTG) staining. After normalizing all the data to the value of CL1 at 48h, it is evident that CL4 cells have higher membrane potential than CL1 at 48h (Fig. S4a), followed by a progressive drop to values lower than CL1 afterwards. Interestingly, the mitochondrial mass/content in these fibroblasts displayed similar alterations over the time (Fig. S4b). These results demonstrate that 48 h after seeding CL4 cells display both a decreased mitochondrial content and activity, followed by similar but lesser trends in the other cell types.

Tissue/cell type	ϕ_M	Ref.
Osteosarcoma cells	0.077	7
Rat renal tubular epithelium	0.14	8
PC-12 cells	0.10	9
Monkey brain cells	0.07	10
Cortical neurons	0.10	11
HeLa cells	0.07	12
Rat hepatoma cell line	0.06-0.10	13
Hamster muscle, glycolytic	0.10	14
Hamster muscle, oxidative	0.20	14
Cardiac muscle myofilaments, human	0.25	15
Cardiac muscle myofilaments, mouse	0.38	15
Fish muscles	0.16-0.37	16,17
Hummingbird flight muscles	0.35	18

Table S1: Experimental estimates of the cell volume fraction, ϕ_M, occupied by mitochondria.

Supplementary Figures

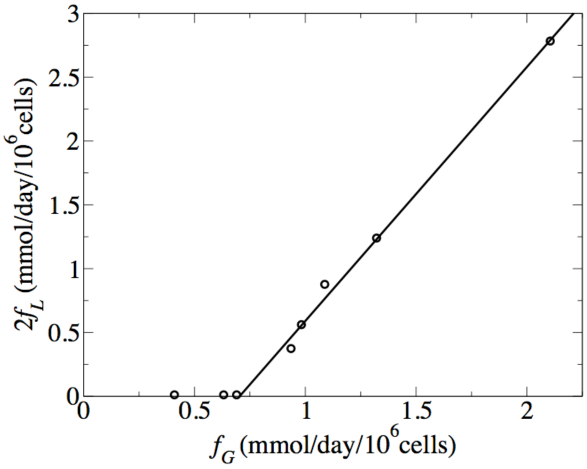

a)

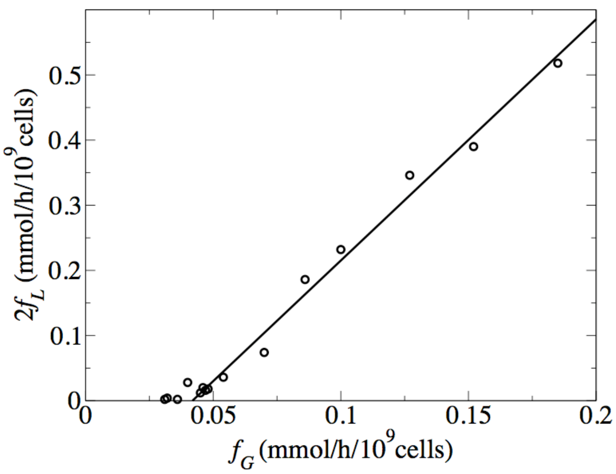

b)

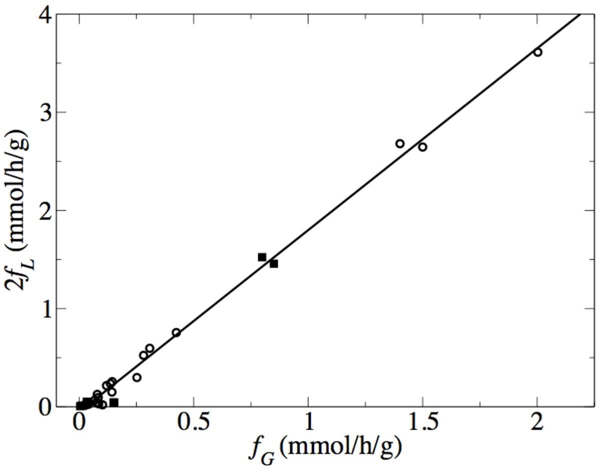

c)

Figure S1: Linear fit to the lactate excretion vs glucose uptake plots.

Lactate excretion rate as a function of the glucose uptake rate for (**a**.) LS mouse cells; (**b.**) hybridoma cells; and (**c.**) a mixture of cancer (circles) and normal (squares) cells. The line represents the best linear fit to the region with a non-zero lactate excretion rate. The glucose uptake rate threshold, f_1, was computed from the ratio between the intercept and the slope, resulting in the values reported in Figure 2e of the main manuscript.

a)

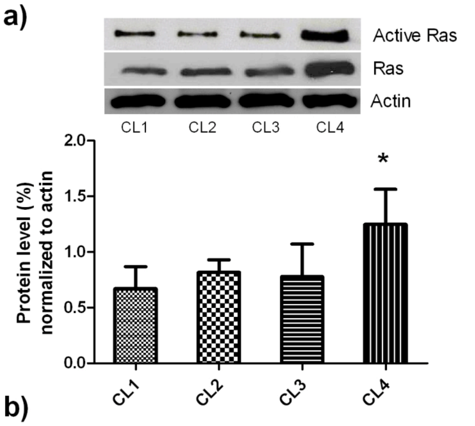

b)

Colony formation	CL1	CL2	CL3	CL4
	0	0	2	453

c)

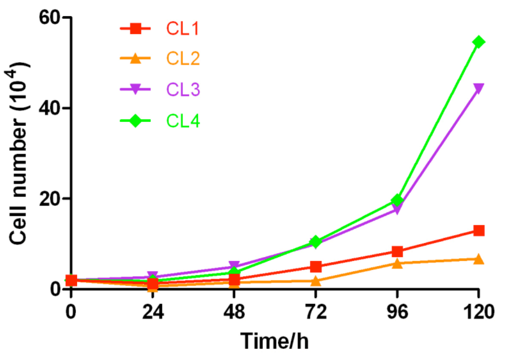

Doubling time	CL1	CL2	CL3	CL4
	28.99537	29.34347	23.8588	19.65457

Figure S2 H-Ras expression, colony formation and growth rate of normal and tumorigenic fibroblasts

(**a.**) Western blot of total H-Ras and active H-Ras in CL1-4 cells. For total H-Ras immunoblots, 50µg protein of each cell line lysates were subjected to 15% Tris SDS-PAGE gel, transferred to PVDF membrane, and immunostained with Ras (Santa Cruz Biotech) and actin-specific monoclonal antibodies (Sigma); all experiments were performed in triplicates and the mean ± SD are shown. *: $p<0.05$. For the Ras activity assay, CL1-4 cell lysates (1mg) were subjected to active Ras pull-down assay (Thermo Scientific) and fifty percent of the eluted samples (25µl, active Ras) were separated by 15% Tris SDS-PAGE gel and immunostained with H-Ras antibody (Thermo Scientific). (**b.**) Soft agar assay. 10,000 cells of each cell type were seeded on 0.35% Top Agar and colonies were stained with 0.005% crystal violet and counted. All experiments were performed in duplicates and the mean numbers are shown in the table; (**c.**) Growth curves of CL1, CL2, CL3 and CL4 cells. For each measurement, continuously cultured fibroblasts, which were cultured in DMEM supplemented with 10% FBS, 25mM glucose and 4mM glutamine in a 37 °C humidified CO_2 incubator, were seeded at 2×10^4 cells in 12-well plates, and cell count data were determined by blinded counting of cells on a standard grid-patterned hemocytometer every 24h.

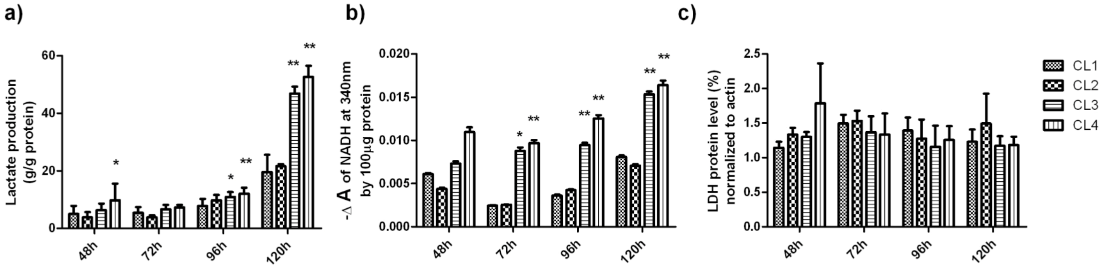

Figure S3 Glycolytic characteristics of normal and tumorigenic fibroblasts

(**a.**) Extracellular lactate levels normalized by the total protein concentration of CL1-4 cells; (**b**.) Relative LDH activity in 100µg protein lysates of CL1-4 cells expressed as the signal drop of NADH fluorescence at 340nm in the first 30s. Mean ± SD of a minimum of three independent experiments are shown. *: $p<0.05$, **: $p<0.01$, ***: $p<0.001$ compared to CL1 at the corresponding time point, in a Student's t-test. (**c**.) Relative LDH protein levels of fibroblasts. 50µg protein of each cell line lysates were subjected to 15% Tris SDS-PAGE gel and immunostained with LDH (Santa Cruz Biotech) and actin-specific monoclonal antibodies (Sigma). It is evident that LDH activities of CL3 and CL4 cells are significantly higher than CL1 and CL2 after 48h.

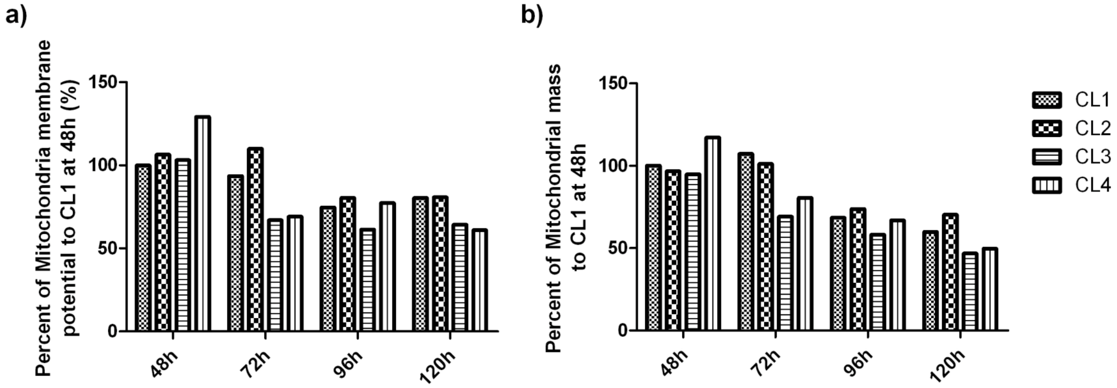

Figure S4 mitochondrial characteristics of normal and tumorigenic fibroblasts

(**a**.) Mitochondrial membrane potential of the indicated cell types are shown, as determined by JC1 staining and subsequent flow cytometry analyses; (**b**.) Mitochondrial content/ mass of the indicated cell types are shown, as determined by MitoTracker Green staining and subsequent flow cytometry analyses. In both panels all values have been normalized to that of CL1 fibroblasts at 48h.

References

[1] Hahn, W.C., et al. (1999) Creation of human tumour cells with defined genetic elements. Nature, 400, 464-468

[2] Cifone, M. A. & Fidler, I. J. (1980) Correlation of patterns of anchorage-independent growth with in vivo behavior of cells from a murine fibrosarcoma. Proc. Natl Acad. Sci. USA 77, 1039-1043.

[3] Vassault A. Lactate Dehydrogenease: UV-method with pyruvate and NADH. In Methods of enzymatic analysis, 3rd ed, 1983. Bergmeyer HU., Ed.; Plenum: New York, Vol. III, p. 118-125.

[4] Wu, J., et al., (1992) Correlation of LDH activity with loss of insect cell viability: an assessment of the LDH assay. Biotechnology Techniques, 6, 335-340.

[5] Ramanathan A, Wang C, Schreiber SL., (2005) Perturbational profiling of a cell-line model of tumorigenesis by using metabolic measurements. Proc Natl Acad Sci U S A. 102, 5992-7.

[6] Hahn, W.C., et al. (2002) Enumeration of the simian virus 40 early region elements necessary for human cell transformation. Mol Cell Biol, 22, 2111-2123.

[7] Singh I, Tsang KY and Ludwig GD (1974) alterations in the mitochondria of human osteosarcoma cells with glucocorticoids. Cancer Res 34, 2946-2952.

[8] Lauschova I et al (2004) Ultrastructural morphometry or renal tubule epithelium in rats treated with conventional amphotericin B deoxycholate or amphotericin B colloidal dispersion. Acta Vet. BRNO 73, 165-169.

[9] Sesso A et al (2004) Morphology of mitochondrial permeability transition: morphometric volumetry in apoptotic cells. The Anatomical Record Part A 281A, 1337-1351.

[10] Bertoni-Freddari C et al (2007) Synaptic and mitochondrial morphometry provides structural correlates of successful brain aging. Ann NY Acad Sci 1097, 51-53.

[11] Yuan H et al (2007) Mitochondrial fission is an upstream and required event for bax foci formation in response to nitric oxide in cortical neurons. Cell Death & Diff 14, 462-471.

[12] Posakony JW, England JM and Attardi G (1977) Mitochondrial growth and division during the cell cycle in HeLa cells. J Cell Biol 74, 468-491.

[13] DiSorbo DM, Paavola LG and Litwack G (1982) Pyrodoxine resistance in a rat hepatoma cell line. Cancer Res 42, 2362-2370.

[14] Sullivan SM and Pittman RN (1987) Relationship between mitochondrial volume density and capillarity in hamster muscles. Am J Physiol Heart Circ Physiol 252, H149-H155.

[15] Barth E, Stammler G, Speiser B and Schaper J (1992) Ultrastructral quantitation of mitochondria and myofilaments in cardiac muscle from 10 different animal species including man. J Mol Cell Cardiol 24, 669-681.

[16] Urschel MR and O'Brien KM (2008) High mitochondrial densities in the hearts of

Antarctic icefishes are maintained by an increase in mitochondrial size rather than mitochondrial biogenesis. J Exp Biol 211, 2638-2646.

[17] O'Brien KM and Sidell BD (2000) The interplay among cardiac ultrastructure, metabolism and the expression of oxygen-binding proteins in Antarctic fishes. J Exp Biol 203, 1287-1297.

[18] Suarez RK, Lighton JRB, Brown GS and Mathieu-Costello O (1991) Mitochondrial respiration in hummingbird flight muscles. Proc natl Acad Sci USA 88, 4870-4873.
